# ICAM-1 is a key receptor mediating cytoadherence and pathology in the *Plasmodium chabaudi* malaria model

**DOI:** 10.1186/s12936-017-1834-8

**Published:** 2017-05-03

**Authors:** Deirdre A. Cunningham, Jing-wen Lin, Thibaut Brugat, William Jarra, Irene Tumwine, Garikai Kushinga, Jai Ramesar, Blandine Franke-Fayard, Jean Langhorne

**Affiliations:** 1 The Francis Crick Institute, London, NW1 1AT UK; 2 Leiden Malaria Research Group, Leiden University Medical Center, 2333 ZA Leiden, The Netherlands

**Keywords:** *Plasmodium chabaudi*, Sequestration, ICAM-1, CD36, Schizont membrane-associated cytoadherence protein, SMAC

## Abstract

**Background:**

Parasite cytoadherence within the microvasculature of tissues and organs of infected individuals is implicated in the pathogenesis of several malaria syndromes. Multiple host receptors may mediate sequestration. The identity of the host receptor(s), or the parasite ligand(s) responsible for sequestration of *Plasmodium* species other than *Plasmodium falciparum* is largely unknown. The rodent malaria parasites may be useful to model interactions of parasite species, which lack the *var* genes with their respective hosts, as other multigene families are shared between the species. The role of the endothelial receptors ICAM-1 and CD36 in cytoadherence and in the development of pathology was investigated in a *Plasmodium chabaudi* infection in C57BL/6 mice lacking these receptors. The schizont membrane-associated cytoadherence (SMAC) protein of *Plasmodium berghei* has been shown to exhibit reduced CD36-associated cytoadherence in *P. berghei* ANKA-infected mice.

**Methods:**

Parasite tissue sequestration and the development of acute stage pathology in *P. chabaudi* infections of mice lacking CD36 or ICAM-1, their respective wild type controls, and in infections with mutant *P. chabaudi* parasites lacking the *smac* gene were compared. Peripheral blood parasitaemia, red blood cell numbers and weight change were monitored throughout the courses of infection. Imaging of bioluminescent parasites in isolated tissues (spleen, lungs, liver, kidney and gut) was used to measure tissue parasite load.

**Results:**

This study shows that neither the lack of CD36 nor the deletion of the *smac* gene from *P. chabaudi* significantly impacted on acute-stage pathology or parasite sequestration. By contrast, in the absence of ICAM-1, infected animals experience less anaemia and weight loss, reduced parasite accumulation in both spleen and liver and higher peripheral blood parasitaemia during acute stage malaria. The reduction in parasite tissue sequestration in infections of ICAM-1 null mice is maintained after mosquito transmission.

**Conclusions:**

These results indicate that ICAM-1-mediated cytoadherence is important in the *P. chabaudi* model of malaria and suggest that for rodent malarias, as for *P. falciparum*, there may be multiple host and parasite molecules involved in sequestration.

**Electronic supplementary material:**

The online version of this article (doi:10.1186/s12936-017-1834-8) contains supplementary material, which is available to authorized users.

## Background

Adhesion of *Plasmodium*-infected red blood cells to endothelial cells lining the microvasculature of tissues of infected individuals (sequestration) has been implicated in the development of several severe malaria syndromes [1, 2]. For the human malaria parasite *Plasmodium falciparum,* molecules known to interact with the endothelium are members of the *Pf*EMP1 family, encoded by the *var* multigene family [3]. These have been well characterized, and indeed multiple endothelial cell receptors for *Pf*EMP1 have been identified (reviewed by [4, 5]). For the other human malaria species lacking *Pf*EMP1, or the rodent malaria species, little is known about the mechanisms of cytoadherence, with respect to either the parasite molecules or the host proteins with which they interact. The genomes of the human and rodent parasite species that lack the *var* family encode several other multigene families, some of which are not represented in *P. falciparum or Plasmodium reichenowi*, and thus rodent malaria can be used as a model to dissect the mechanisms involved [6, 7]. For *Pf*EMP1 interaction with the host molecules, CD36 and ICAM-1 were identified early [8], subsequently supported by both data from field studies and experimental models (reviewed by [9]). In addition to the restriction of blood flow by occlusion of the microvessels, adherent parasites may also be the focus of an inflammatory response.

CD36 is a class B scavenger receptor expressed on the surface of microvascular endothelium and many other cells and has a role in inflammation, angiogenesis and lipid metabolism (reviewed by [10]). It can bind to clinical isolates of *P. falciparum-*infected red blood cells (iRBC) in vitro, however most clinical studies show an association of CD36 binding with uncomplicated malaria rather than severe disease (reviewed by [9, 11]). CD36 is also involved in internalization and phagocytosis of *P. falciparum* iRBC, suggesting a role in parasite clearance [12]. In the experimental model of *Plasmodium berghei* ANKA in mice, sequestration of iRBCs in lungs and adipose tissue in vivo, is partially dependent on CD36 [13], and this process is thought to be a major contributor to lung pathology in the *P. berghei* ANKA model [14].

The cell-surface receptor, ICAM-1, has been shown both by in vitro binding assays and by field studies to be important for cytoadherence of *P. falciparum* although the relationship with disease outcome is unclear. In some studies ICAM-1 expression has been associated with severe falciparum malaria [15] and more severe infections of children [16], whereas a negative correlation with disease was found in a Malawian childhood malaria study [17]. In addition, in vitro binding of *Plasmodium vivax* iRBCs to ICAM-1 was demonstrated in static and flow assays [18, 19]. ICAM-1 expression on vascular endothelium is upregulated in experimental models of cerebral malaria *(P. berghei* ANKA*)* and infections of mice deficient in ICAM-1 expression (*icam1*−*/*−) show improved survival [20, 21] and reduced sequestration in the alveolar capillaries of the lung [21]. Upregulation of ICAM-1 expression has also been observed on macrophages in *P. berghei* ANKA infections, and it has been suggested that binding of *P. berghei-*iRBC to macrophages could restrict venous blood flow thereby contributing to the cerebral symptoms of this model of experimental cerebral malaria [22]. In the *Plasmodium chabaudi* model an increased expression of ICAM-1 has been associated with a high parasite liver burden [23].

Sequestration has been described for other species of *Plasmodium* including *P. vivax* and the rodent-infecting species, which do not have the *var* genes encoding *Pf*EMP1 [13, 24–26]. However, in these cases the parasite ligands responsible for cytoadherence are not known.

In the rodent malaria parasite, *P. berghei* ANKA, a large proteomic screen of parasite membrane proteins identified a 17kD schizont membrane-associated protein (SMAC) [27], which was exported to the iRBC cytoplasm. *Plasmodium berghei* ANKA parasites lacking SMAC (Δ*smac*) exhibited a different cytoadherence pattern from that of wild-type parasites, with a high number of mature schizonts in the peripheral blood and reduced accumulation in adipose tissue, suggesting that SMAC, although not on the iRBC surface, is implicated in some way in sequestration. In the absence of CD36, both wild type (wt) and Δ*smac* mutant parasites exhibited similar distribution patterns, indicating that Δ*smac* might be involved in the CD36-mediated sequestration [27].

In order to determine whether adherence to ICAM-1 or to CD36 and the involvement of SMAC was applicable to other rodent malarias, the effect of the lack of these molecules on a *P. chabaudi* blood-stage infection of C57BL/6 mice was investigated. The advantage of the *P. chabaudi* infection is that it maintains a naturally synchronous asexual developmental cycle [28], and sequestration, mainly in liver and lung, can be observed both by removal of schizonts from peripheral blood and by sequestration in tissues at a defined time of the day [29]. These data show that the CD36 receptor in mice and the lack of SMAC in the parasite, unlike for *P. berghei*, have limited impact on sequestration and pathology. By contrast, the lack of the adhesion molecule ICAM-1 reduces parasite burden in liver and spleen, and reduced the clinical signs of an acute *P. chabaudi* infection.

Therefore it appears that with the rodent malarias there may be multiple host and parasite molecules involved in sequestration. This may well be the case for other human malarias, such as *P. vivax*.

## Methods

### Mice

Female C57BL/6 mice aged 6–8 weeks from the Specific Pathogen Free unit at the Francis Crick Institute Mill Hill Laboratory were housed under reverse light conditions (light 19.00–07.00, dark 07.00–19.00 GMT) at 20–22 °C, and had continuous access to mouse breeder diet and water. Body weight was calculated relative to a baseline measurement taken before infection and erythrocyte density was determined on a VetScan HM5 haematology system (Abaxis). This study was carried out in accordance with the UK Animals (Scientific Procedures) Act 1986, and was approved by The Francis Crick Institute Ethical Committee.

CD36-null C57BL/6 (*cd36*−*/*−*)* mice were a kind gift from Maria Febbraio, Lerner Research Institute, Cleveland Clinic, Ohio, USA [30]. CD36-null mice and wt *(cd36*+*/*+*)* control lines derived from littermates were bred in-house as above. ICAM-1 null [B6.129S7-*Icam1tm1Bay*/J] mice [31] (a gift from Arthur Beaudet, Baylor College of Medicine, Houston, TX, USA) were generated from a 129 ES line into a 129× C57BL/6 backcrossed at least seven generations to in-house C57BL/6. Control lines wt (*icam*-*1*+*/*+) were derived from littermates and bred in-house as above.

### Parasites and mosquitoes

A cloned line of *P. chabaudi chabaudi* AS was originally obtained from David Walliker, University of Edinburgh, UK and subsequently passaged through mice by injection of iRBCs at the MRC National Institute for Medical Research, UK and cryopreserved as described [32]. Unless otherwise indicated, infections were initiated by intraperitoneal (ip) injection of 10^5^ iRBC derived from cryopreserved stocks. The course of infection was monitored on Giemsa-stained thin blood films by enumerating the percentage of iRBC with asexual parasites (parasitaemia). Where indicated, infections were initiated by infection with 10^5^ iRBC derived from parasites cryopreserved immediately after transmission through *Anopheles stephensi* mosquitoes (recently transmitted parasites). Mosquitoes were bred, housed and transmitted as previously described [33]. The asexual developmental stage of the parasites in the peripheral blood was determined by performing differential counts (rings, trophozoites, schizonts) on Giemsa-stained thin blood films prepared from tail blood collected at specific time points throughout the dark light cycle (GMT 09:00, 13:30,18:00), day 7 post-infection, when the number of parasites in the blood was at a level which allowed enumeration of the various stages and cells infected with multiple parasites were not found.

*Plasmodium chabaudi* AS expressing luciferase under the control of the *P. berghei* constitutive promoter *eef1a* (*Pc*ASluc_230p_) was generated by transfection with the construct pPc-LUC230p targeting neutral *P230p* (PCHAS_0308200) locus. The construct pPc-LUC230p was modified from pPc-LUCCAM [29] by replacing *the P. chabaudi* SSU targeting region with *230p* targeting region, Chab03 277001-278950, generated by gene synthesis (Genewiz LLC, NJ, USA) and linearized with *apa1* prior to transfection [34]. *Plasmodium chabaudi* AS parasites lacking expression of SMAC (*Pc*ASΔ*smac*) were generated by replacing the *P. berghei smac* (PBANKA_0100600) targeting region [27] with the equivalent SMAC region of *P. chabaudi* (PCHAS_0101300; PCHAS_01_v3 52494-53200 and 53700-54284). Δ*smac* parasites expressing luciferase constitutively were subsequently generated using a modified construct (*Pc*ASΔ*smacEFluc*) whereby *eef1a* luciferase was inserted within the targeting region at *Eco*RV/*Eco*R1 sites (Additional file 1a). Both Δ*smac* and Δ*smacEFluc* constructs were digested with Kpn1/SacII prior to transfection. Transfection and cloning of transgenic *P. chabaudi* parasites were performed as described previously [35], and integration was verified by PCR (see Additional files 1b, 2) and by Southern blot analysis of chromosomes separated by pulsed field gel (PFG) as described [36], and Additional file 1c. Serial dilutions (10^3^–10^5^) of *Pc*ASluc_230p_ (EFlucWT) iRBC and *Pc*ASΔ*smacEFluc* iRBC were performed and luciferase activity assayed as described below (Additional file 1d).

### In vivo imaging and luciferase assay

Mice were infected intraperitoneally with 10^5^ iRBC infected with *Pc*ASluc_230p_ or *Pc*ASΔ*smacEFluc* parasites; and at each time point 2 μL of heparinized tail blood was collected before the time of sequestration. Bioluminescence was assessed with the Luciferase Assay System (Promega) according to the manufacturer’s protocol and quantified with the TECAN Safire2 plate reader and Magellan software (Tecan). Under these conditions, bioluminescence intensity is proportional to the amount of parasites in the blood volume, which reflects the total parasite load before sequestration. At the time of maximum sequestration (12.00–14.00 h GMT, reverse light), d-luciferin (150 mg/kg, Caliper Life Sciences) was injected subcutaneously 5 min before whole body and organ imaging. Mice were terminally anaesthetized and systemically perfused by intracardiac injection of 10 mL PBS [29]. The brain, lungs, liver, spleen, left kidney, and gut were removed immediately and luciferase activity assessed using in vivo Imaging System IVIS Lumina (Xenogen), with a 10-cm field of view, a binning factor of 4, and an exposure time of 10 s. Bioluminescence (p/s) was quantified with the software Living Image (Xenogen) by adjusting a region of interest to the area of each organ. The use of luciferase activity in the organs as a measure of sequestering parasites was validated previously using light and electron microscopy [29]. An increase in the percentage parasitaemia within the microvessels of lungs and liver at the time of schizogony was also demonstrated in this previous study using a quantitative analysis of tissue sections.

To account for the influence of total parasite load on the number of parasites sequestered in the organs, bioluminescence in the organs was normalized to total parasite load. This value was taken to reflect parasite burden in the whole body. The total flux (p/s) for each organ was normalized to blood luciferase activity (09:00 h, trophozoite stage parasites, 2 μL blood) to facilitate the comparison of parasite load in tissues taken from infected animals with different levels of blood parasitaemia.

### Statistical analysis

Data are shown as means and SEM. The non-parametric Mann–Whitney U test was used and p values below 0.05 were considered as statistically significant.

## Results

### Absence of ICAM-1, but not CD36, ameliorates acute blood-stage pathology and reduces tissue sequestration of *Plasmodium chabaudi*

The effect of binding to ICAM-1 or CD36 in pathology and sequestration in vivo during a blood-stage infection with *P. chabaudi* in mice was investigated.

**Fig. 1:**
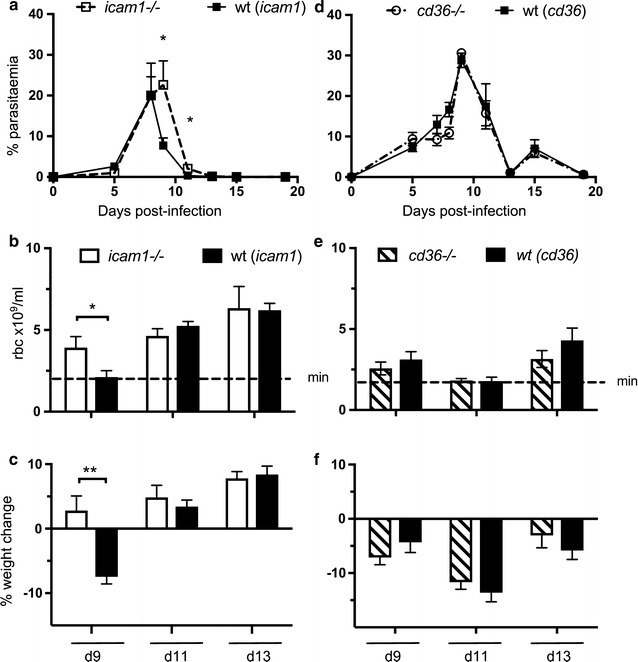
The ICAM-1 receptor and not the CD36 receptor impacts acute stage pathology of *Plasmodium chabaudi* AS infections. Peripheral blood parasitaemia (**a**, **d**) anaemia (**b**, **e**) and weight loss (**c**, **f**) were compared in serially blood passaged *P. chabaudi* AS infections of *icam*-*1*−*/*− mice (**a**–**c**), *cd36*−*/*− mice (**d**–**f**) and their controls. Infections in mice lacking the ICAM-1 receptor resulted in less severe weight loss (day 9, **p < *0.01*) and anaemia (day 9, *p < *0.05*), yet parasite levels in peripheral blood were higher at this time point (*p < *0.05*), compared to infections of wt mice. Infections in *cd36*−/− animals showed similar anaemia, weight loss and peripheral blood parasitaemia as infections of wt animals throughout the infection (n = 6)

Mice lacking ICAM-1 (*icam1*−*/*−*)* infected by i.p. injection of 10^5^ *P. chabaudi* iRBC developed a higher parasitaemia in peripheral blood (days 9 and 11) (*p* < *0.05*), (Fig. 1a) yet exhibited less severe pathology, both in terms of anaemia (day 9, *p* < *0.01*) (Fig. 1b) and weight loss (day 9, *p* < *0.05*) (Fig. 1c) during the acute infection, compared with wild-type (wt) C57BL/6 mice.

By contrast, the lack of CD36 did not impact significantly on the course of a primary acute infection or on the accompanying anaemia and weight loss. Parasitaemia (Fig. 1d), anaemia (Fig. 1e) and weight loss (Fig. 1f) were similar between *cd36*−*/*− and wt controls and for both groups of mice the infections resolved with 100% survival.

**Fig. 2:**
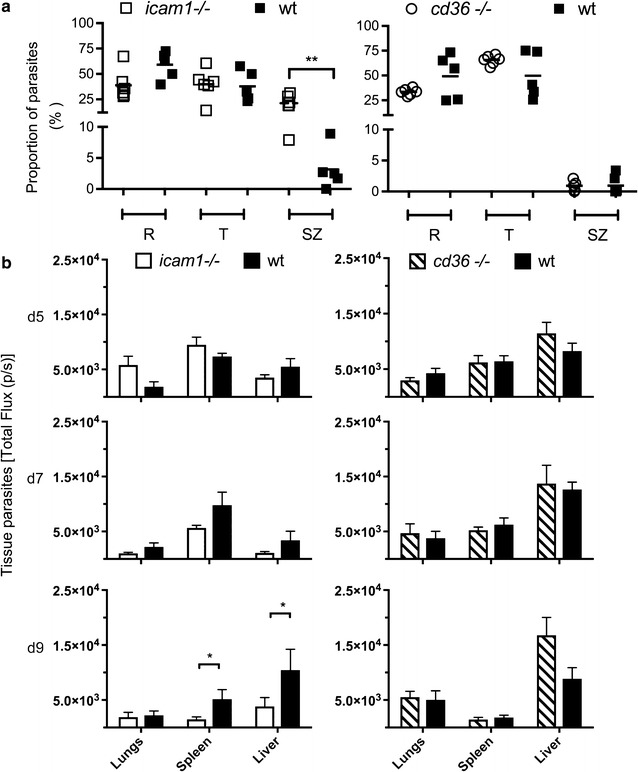
The ICAM-1 receptor, but not the CD36 receptor, plays a role in sequestration. **a** A higher proportion of schizonts (Sz) were seen in peripheral blood of *icam*-*1*−*/*− mice at the time of schizogony (GMT 13:30, day 7 post-infection, **p < *0.01*) compared to wild-type mice. Proportions of rings (R) and trophozoites (T) were similar to control wt mice. The proportion of peripheral blood schizonts seen in peripheral blood of *cd36*−*/*− mice was similar to that of control wt mice (n = 6). **b** Tissue parasites were reduced in spleen and liver of *icam*-*1*−*/*− mice, after the infection peak (day 9, *p < *0.05*). At earlier time points (day 5 or 7 post-infection) no significant differences in tissue parasites were seen. Infections of *cd36*−*/*− mice showed similar sequestration patterns to those observed in wild type animals throughout the infection (n = 5)

To investigate whether the lack of CD36 or ICAM-1 influenced parasite sequestration or accumulation of iRBC in different organs, the distribution of parasite life-cycle stages in peripheral blood was first determined, as a measure of withdrawal of mature forms into tissues and organs. The distribution of the life cycle stages in peripheral RBC of *P. chabaudi*-infected *icam1*−*/*− mice but not *cd36*−*/*− mice, was different from that of wt C57BL/6 mice. There was a greater proportion of schizonts present in the blood at the time when RBC infected with mature stage parasites have normally withdrawn from peripheral blood of wt mice [29], (*p* < *0.01*) (Fig. 2a).

*Plasmodium chabaudi* AS expressing luciferase constitutively (*Pc*AS*luc*) [29] was used to infect *cd36*−*/*−, *icam1*−*/*− and their respective wt control mice in order to measure parasite load in spleen, liver, lungs, kidney, gut and brain at days 5, 7 and 9 post-infection. The amount of tissue parasites was reduced in spleen and liver of *icam1*−*/*− mice compared to infected wt mice after the infection peak (day 9, *p* < *0.05*). Differences in the level of tissue parasites in the lungs, spleen and liver at the earlier time points (days 5 or 7) did not reach significance (Fig. 2b) while similar levels of tissue parasites were seen for kidney or gut in infections of *cd36*−*/*−, *icam1*−*/*−, and their respective controls, at all three time points measured (Additional file 3). Kidney was not an important sequestration site and in this and all subsequent experiment series described here, similarly negligible levels of tissue parasites were seen in kidney and gut (Additional file 3). Also, as previously observed [29], brain was uniformly negative in all experiments and thus has not been included here. In spite of the increased number of mature parasites remaining in the blood, *icam1*−*/*− mice were less sick.

In contrast to the changes observed in sequestration in the *icam1*−*/*− mice, the patterns observed in infected *cd36*−*/*− mice were similar to those of wt animals (Fig. 2b). As *P. chabaudi* AS iRBCs still sequester in the absence of CD36 it is clear that CD36 is not a major receptor for tissue cytoadherence in this experimental model of malaria.

**Fig. 3:**
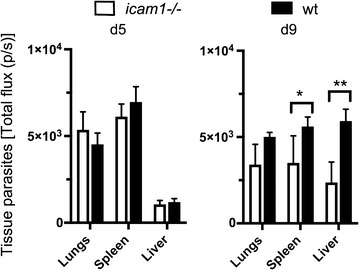
Interaction with the ICAM-1 receptor is maintained after vector transmission of the parasite. Tissue parasites were reduced in spleen (*p < *0.05*) and liver (**p < *0.01*) of mice infected with recently mosquito-transmitted *P. chabaudi* AS parasites at day 9 post-infection but not at the earlier time point (day 5) (n = 6)

To investigate whether decreased tissue sequestration was also the case in the blood stage infection that develops after transmission of the parasite by mosquito or by inoculation of recently mosquito-transmitted parasites [33], both groups of mice were inoculated with 10^5^ *P. chabaudi* AS iRBC, which had recently been transmitted through mosquitoes and the parasite load in the tissues at the time of schizogony was measured. Imaging of perfused tissues showed that tissue parasites were significantly reduced in spleens (*p* < *0.05*) and livers (*p* < *0.01*) of *icam1*−*/*− mice infected with recently mosquito-transmitted iRBC at day 9 post-infection but not at the earlier time point (day 5), while differences in sequestration in the lungs did not reach significance (Fig. 3).

### The absence of SMAC does not affect tissue sequestration of *Plasmodium chabaudi*

**Fig. 4:**
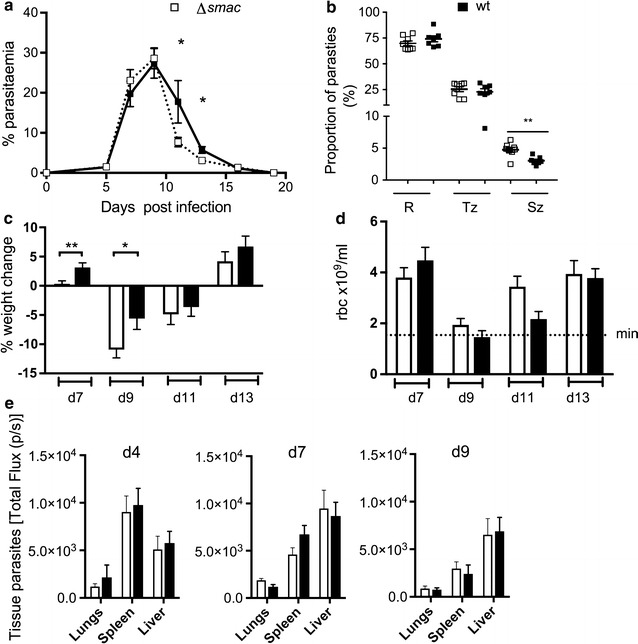
Acute stage pathology increases but parasite tissue sequestration is unchanged in *Plasmodium chabaudi* parasites lacking SMAC. **a** Wild-type parasites attained a higher peripheral blood parasitaemia after the peak of infection than mice infected with *Pc*ASΔ*smac* parasites (*p < *0.05*) (n = 9). **b** The proportion of schizonts (SZ) appearing in the peripheral blood increased, for Δ*smac* mutants compared to wild-type parasites (**p < *0.01*), enumerated at the time of schizogony (GMT 13:30, day 7 post-infection). Proportions of rings (R) and trophozoites (T) were similar to control wt parasites (n = 6). **c** The mutant parasites showed more severe weight loss around the infection peak (day 7, **p < *0.01*; d9, * *p* < *0.05*) (n = 9). **d** Red blood cell loss was similar to that observed in mice infected with wt parasites (n = 9). **e** Δ*smac* and wt parasites accumulated at similar levels in the liver, lungs, and gut both pre (day 4), during (day 7) and post (day 9) infection peak (n = 6)

It has previously been reported that *P. berghei* ANKA parasites lacking SMAC (Δ*smac*) exhibited a high number of mature schizonts in the peripheral blood and reduced parasite accumulation in adipose tissue, suggesting that SMAC, although not on the iRBC surface, is implicated in some way in sequestration [27]. To see whether this molecule could be similarly involved in sequestration of *P. chabaudi* iRBC, a gene deletion mutant of the *P. chabaudi* orthologue of the *smac* gene (PCHAS_0101300) in *P. chabaudi* AS, *Pc*ASΔ*smac* was generated (Additional file 1), and the course of infection, weight and red cell concentration was compared with wt parasites. Infection with wt parasites attained a slightly higher peripheral blood parasitaemia after the peak of infection than the mutant parasites (*p* < *0.05*) (Fig. 4a). Although the parasitaemias were essentially similar for most of the acute stage there was a greater proportion of schizonts in peripheral blood for the *smac*−*/*− mutants (Fig. 4b) (*p* < *0.01*), suggesting that sequestration in the tissues was indeed slightly compromised. Mice infected with *PcAS*Δ*smac* parasites lost significantly more weight around the infection peak (day 7, *p* < *0.01*; day 9, *p* < *0.05*) (Fig. 4c). Red blood cell loss (Fig. 4d) was similar in both groups.

The *Pc*ASΔ*smacEFluc* mutant was generated, in which the *smac* gene was deleted and at the same time the expression of luciferase was introduced, (Additional file 1), and the impact of the lack of this gene on sequestration was investigated. The *Pc*AS*Luc* and *Pc*ASΔ*smacEFluc* parasites emitted the same amount of light per parasite (RLU) (Additional file 1d) thus allowing comparison of parasite load in the various organs by total flux. Despite the small but significant increase in the proportion of schizonts in peripheral blood of mice infected with *Pc*ASΔ*smac,* (day 7) (Fig. 4b), imaging of luciferase-tagged parasites showed no significant difference in tissue parasites between infections with the mutant and wild-type parasites, at days 4, 7 and 9 post-infection with respect to all organs measured, although there was a trend towards a reduced splenic parasite load in the mutant parasites on day 7 (Fig. 4e; Additional file 4).

Together these data suggest that the ICAM-1 molecule and not CD36 is an important cytoadherence receptor for *P. chabaudi* and further supports the hypothesis that cytoadherence is important for the development of pathology.

## Discussion

ICAM-1 and CD36 are strong candidate receptors for interactions with iRBC containing mature *Plasmodium* parasites within the microvasculature at the time of sequestration, in both human infections and rodent model infections, and for the associated development of severe malaria pathologies. As species-specific differences in human malaria manifest themselves in distinctions in the nature and severity of the pathological syndromes developed, such differences are also seen in experimental models of malaria where the different host and parasite combinations exert similar influences [9].

The data presented here suggest that the ICAM-1 molecule is an important cytoadherence receptor for *P. chabaudi* in the spleen and liver, and further supports the hypothesis that cytoadherence is important for the development of pathology. Infected mice lacking the ICAM-1 receptor are less anaemic and lose less weight than mice with control infections, despite developing a higher peripheral blood parasitaemia which remained high for a longer time. The increase in parasitaemia was mirrored by a decrease in accumulation of mature parasites in spleen and liver. The lack of ICAM-1 has a marked effect on tissue sequestration in spleen and liver. It has previously been shown that sequestration in the livers of wild-type animals and the associated liver damage and increased liver pathology are at a maximum at day 9, when there is also a considerable host response [29]. It is likely that local production of cytokines in the tissues in response to sequestering parasites would further enhance the expression of ICAM-1 in the wild-type host, contributing both to weight loss and the development of anaemia. Although clearly the lack of ICAM-1 modulates the infection in vivo, the capacity of *P. chabaudi* iRBC to bind to cell lines expressing different recombinant receptors in vitro would give more direct evidence of the relevant host/parasite interactions. Such binding has been shown for the human malaria species *P. vivax* and *P. falciparum* [18, 19]. *Plasmodium berghei* ANKA infections with mutant parasites show reduced sequestration in lungs and adipose tissue and this is accompanied by a higher splenic parasite load, which could lead to more effective clearance of the parasites [27, 37]. These authors show that the parasite mutants either develop lower peripheral blood parasitaemia (SBP1, MAHRP) or can persist to the same degree as wild-type parasites (SMAC) in splenectomized mice. Here, in infections of *icam1*−*/*− mice, reduced parasite sequestration in the liver occurs concomitantly with a reduced load in the spleen, thus peripheral blood parasitaemia increases and parasite clearance is delayed. Thus, the impaired ability to sequester rather than high parasitaemia per se has the greater impact on the health status of the mouse. As it has previously been shown that parasite sequestration in the liver is accompanied by tissue damage and reduced liver function [29], it is possible that such damage is abrogated when sequestration is reduced. Although the possibility that uptake of parasitized cells by monocytes and macrophages of the reticuloendothelial system could contribute to the total measured luciferase activity in the organs here cannot be completely ruled out, this approach was validated previously using light and electron microscopy and parasite quantitation at the time of schizogony.

Although ICAM-1 has been shown to impact sequestration of *P. berghei* ANKA iRBC in alveolar capillaries [21], *P. chabaudi* iRBC sequestration in the lungs was unaffected by ICAM-1. The level of ICAM-1 mRNA is increased in the livers of *P. chabaudi* AS-infected mice [23] and in addition, liver sequestration is reduced in the absence of IFNγR signaling [29]. It is possible that the IFNγR signaling effect is mediated partially via ICAM-1 as IFNγ upregulates the expression of ICAM-1 on endothelial cells [38]. The observed reduced liver sequestration is also seen after transmission through the mosquito, where parasites in both the spleen and liver are reduced compared to controls. This finding of a role for ICAM-1 in sequestration of *P. chabaudi* parasites is in line with the observation that *P. vivax*-infected erythrocytes exhibit enhanced binding to ICAM-1 in vitro, compared to CD36 or to the untransfected control CHO cells [18]. This group found that treatment with anti-VIR antibodies abrogated the binding of *P. vivax*-iRBC to human lung endothelial cells. The predominance of ICAM-1 cytoadherence is also supported by a later study using *P. falciparum* transgenic lines expressing *P. vivax* VIR proteins showing binding of a specific VIR protein to multiple host receptors in vitro under static conditions yet binding to ICAM-1 alone was maintained under flow conditions [19]. For *P. falciparum*, domains of the variant *Pf*EMP1 proteins responsible for interacting with specific host receptors, notably CIDRα1 for EPCR and CIDRα2-6 for CD36 and DBL2β for ICAM-1 [39–41] have been extensively studied. By contrast, the parasite ligand(s) involved in *P. vivax* sequestration are unknown and the relationship to pathology is unclear [42]. The differences in the relative importance of the receptors involved in sequestration, shown here for *P. chabaudi*, compared to *P. falciparum,* may be due to the absence of *var* and the involvement of a different set of parasite ligands, encoded by one of the other multigene families, shared by *P. vivax* and the rodent malaria parasites.

It has previously been shown that *P. chabaudi* iRBC accumulate in the spleen early in infection and later sequester in liver and lung, and we observed damage to liver, lung and kidney [29]. Experiments with transgenic *P. falciparum* lines engineered to express VIR proteins (encoded by the *pir/vir* multigene family) also suggest a stronger interaction with ICAM-1, as under physiological or flow conditions these lines adhere to ICAM-1 but not to CD36 or CSA [19]. Mosquito transmission of *P. chabaudi* AS alters parasite gene expression including upregulation of expression of many *pir/cir* genes, [43]. As the effect of lack of ICAM-1 on tissue accumulation (spleen and liver) is a little more pronounced after the parasite has completed the full developmental cycle, it is possible that expression of the parasite ligands involved in binding to ICAM-1 may be increased after transmission through the mosquito.

The lack of CD36 had no effect on sequestration or pathology in *P. chabaudi* infections, although infected erythrocytes have previously been shown to bind to recombinant human CD36 in vitro [44]. This is in contrast to the *P. berghei* ANKA model, where lack of CD36 afforded no protection from cerebral pathology but sequestration was reduced in lungs and adipose tissue [13] and lung pathology, as manifested by vascular leakage, was reduced [14, 45]. However, a recent study of lung pathology in this model used mouse bone-marrow chimeras to show that endothelial damage was greatest when CD36 was expressed on endothelial cells, suggesting that the presence of CD36 on trafficking cells may result in enhanced parasite clearance and hence offer some protection from endothelial damage [14]. This could explain why in this study infection in the complete *cd36*−*/*− mouse KO is no different from that of wild-type mice as the two roles of CD36 could exert a balancing effect.

Interactions of endothelial cell receptors with parasite ligand(s) may be highly specific, as for CSA [46] which interacts with a very limited number of *Pf*EMP1 proteins, or more promiscuous, in the case of CD36 [16]. Receptors may also interact synergistically with a co-receptor. It has been suggested that for *P. falciparum,* ICAM-1 and EPCR may interact to promote binding to brain endothelial cells (where CD36 expression is much lower), while ICAM-1 and CD36 may act as co-receptors promoting binding in other vascular beds (e.g., lungs). So the relative importance of a particular receptor or receptor combination may be dependent on the vascular niche (e.g., brain *vs* lung *vs* placenta) [47]. However, the lack of an effect of CD36 on sequestration of pathology of *P. chabaudi* infections, in spite of a capacity to bind human CD36, [44] may indicate receptor redundancy rather than synergy.

Significantly more mature schizonts were observed in the peripheral blood at the time of schizogony (day 7 post-infection) in infections with *P. chabaudi* Δ*smac* parasites. However this was not mirrored by any changes in parasite accumulation in the spleen or sequestration in the lungs, liver or other tissues. This result contrasts with that of mice infected with *P. berghei* ANKA Δ*smac* parasites, which exhibited an increased parasite load in the spleen of mice and reduced load in lungs and fat of the same mice [27]. However, *P. berghei* ANKA Δ*smac* parasites also demonstrated reduced CD36-mediated cytoadherence, whereas we could find no evidence of CD36-mediated cytoadherence or any associated pathology in the *P. chabaudi* model. Thus, the lack of a strong effect of *P. chabaudi Δsmac* parasites on tissue sequestration would suggest that the effect is specific to the particular receptor involved in the cytoadherence process. Two further *P. berghei* ANKA gene deletion mutants (SBP1 and MAHRP1) that exhibit reduced sequestration phenotypes have recently been generated and these also show reduced binding to CD36 [37]. It would be of interest to determine whether these molecules could play a role in CD36 independent sequestration, as seen in this *P. chabaudi* infection.

## Conclusions

Absence of ICAM-1, but not CD36, ameliorates acute blood-stage pathology and reduces tissue sequestration of *P. chabaudi.* Interaction with the ICAM-1 receptor is maintained after vector transmission of the parasite. The absence of SMAC results in some increased acute stage pathology does not affect tissue sequestration of *P. chabaudi.* These data suggest that the ICAM1 molecule is an important cytoadherence receptor for *P. chabaudi*. As tissue cytoadherence is reduced but not abolished it is likely that other receptors are also involved.

## Additional files

**Additional file 1.** a) Constructs used to generate *P. chabaudi smac* mutants: (i) Δ*smac*; (ii) Δ*smacEFluc.* b) PCR verification of insertion into the SMAC locus. Integration of the plasmid was verified with primer set P1/P2 and the loss of the wild type locus is shown using primer set P2/P3. Lanes 1–5 contain samples from parasites transfected with Δ *smac* (1), Δ*smacEFluc* (2), wild-type parasites (3–4), water control (5). c) Integration of the plasmids into chromosome 1. PFG separated chromosomes hybridized with a 3′UTR pbdhfr/ts probe show insertion of the plasmid into chromosome 1: *P. chabaudi* wild-type DNA (1), Δ*smacEFluc* (2–3), The probe also hybridizes to the endogenous *P. chabaudi dhfr* locus on chromosome 7. d) Relative light emission levels are similar for Δ*smac* and Δ*smacEFluc* parasites.

**Additional file 2.** PCR primers for verification of integration.

**Additional file 3.** Similar levels of tissue parasites were seen for kidney or gut in infections of *icam1*−*/*−, *cd36*−*/*−, and their respective controls, at days 5, 7 and 9 post-infection.

**Additional file 4.** Similar levels of Δ*smac* and wild-type parasites accumulated in kidney and gut at days 4, 7 and 9 post-infection (n = 6).
