# Supplementary figures and images for: ICAM-1 is a key receptor mediating cytoadherence and pathology in the Plasmodium chabaudi malaria model
Source: Malar J. 2017 May 3;16:185. doi: 10.1186/s12936-017-1834-8 (PMC5415785; doi:10.1186/s12936-017-1834-8)

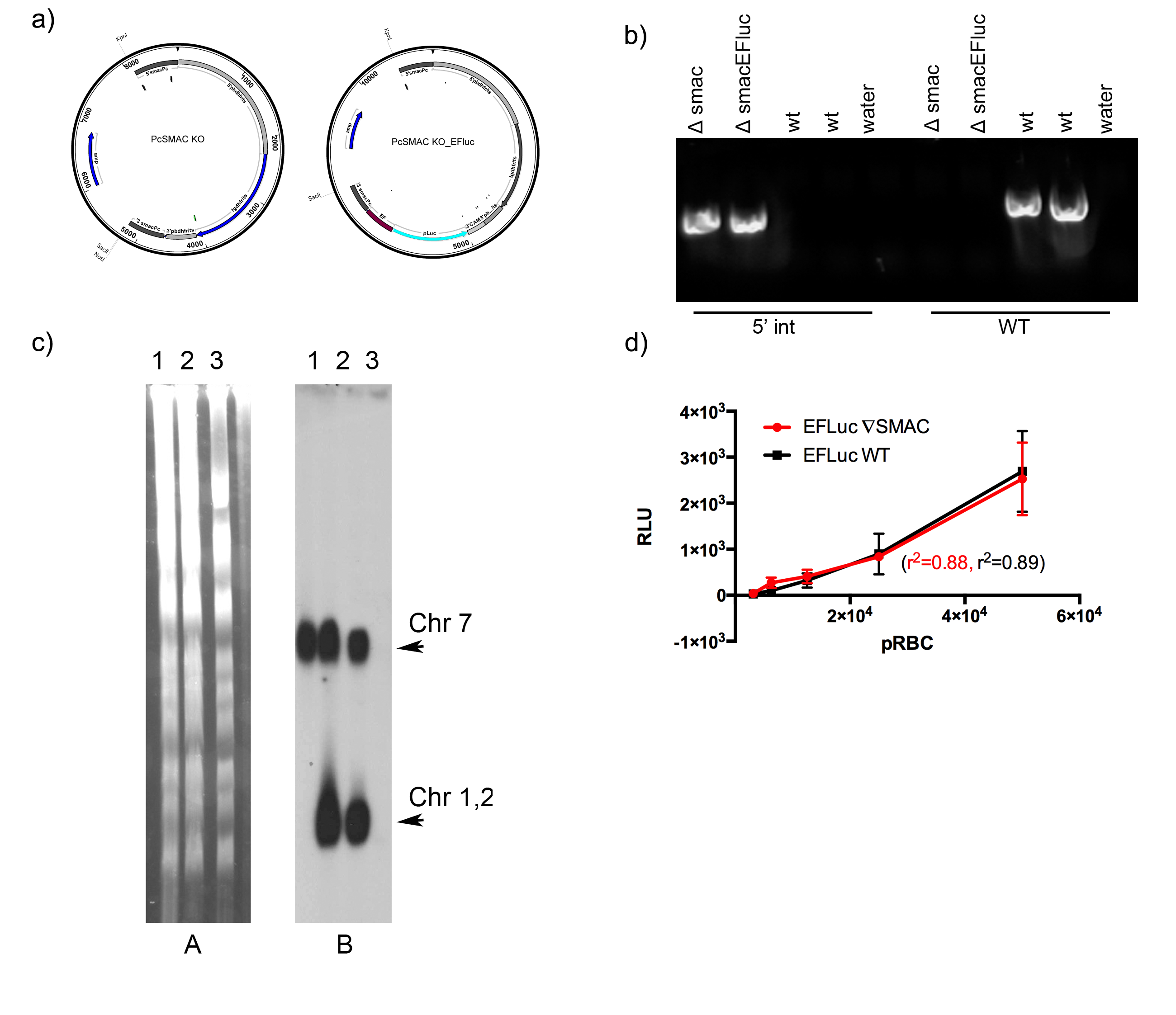

Supplement: Supplementary file 1 — Additional file 1. a) Constructs used to generate P. chabaudi smac mutants: (i) Δsmac; (ii) ΔsmacEFluc. b) PCR verification of insertion into the SMAC locus. Integration of the plasmid was verified with primer set P1/P2 and the loss of the wild type locus is shown using primer set P2/P3. Lanes 1–5 contain samples from parasites transfected with Δ smac (1), ΔsmacEFluc (2), wild-type parasites (3–4), water control (5). c) Integration of the plasmids into chromosome 1. PFG separated chromosomes hybridized with a 3′UTR pbdhfr/ts probe show insertion of the plasmid into chromosome 1: P. chabaudi wild-type DNA (1), ΔsmacEFluc (2–3), The probe also hybridizes to the endogenous P. chabaudi dhfr locus on chromosome 7. d) Relative light emission levels are similar for Δsmac and ΔsmacEFluc parasites. [file 12936_2017_1834_MOESM1_ESM.tif]

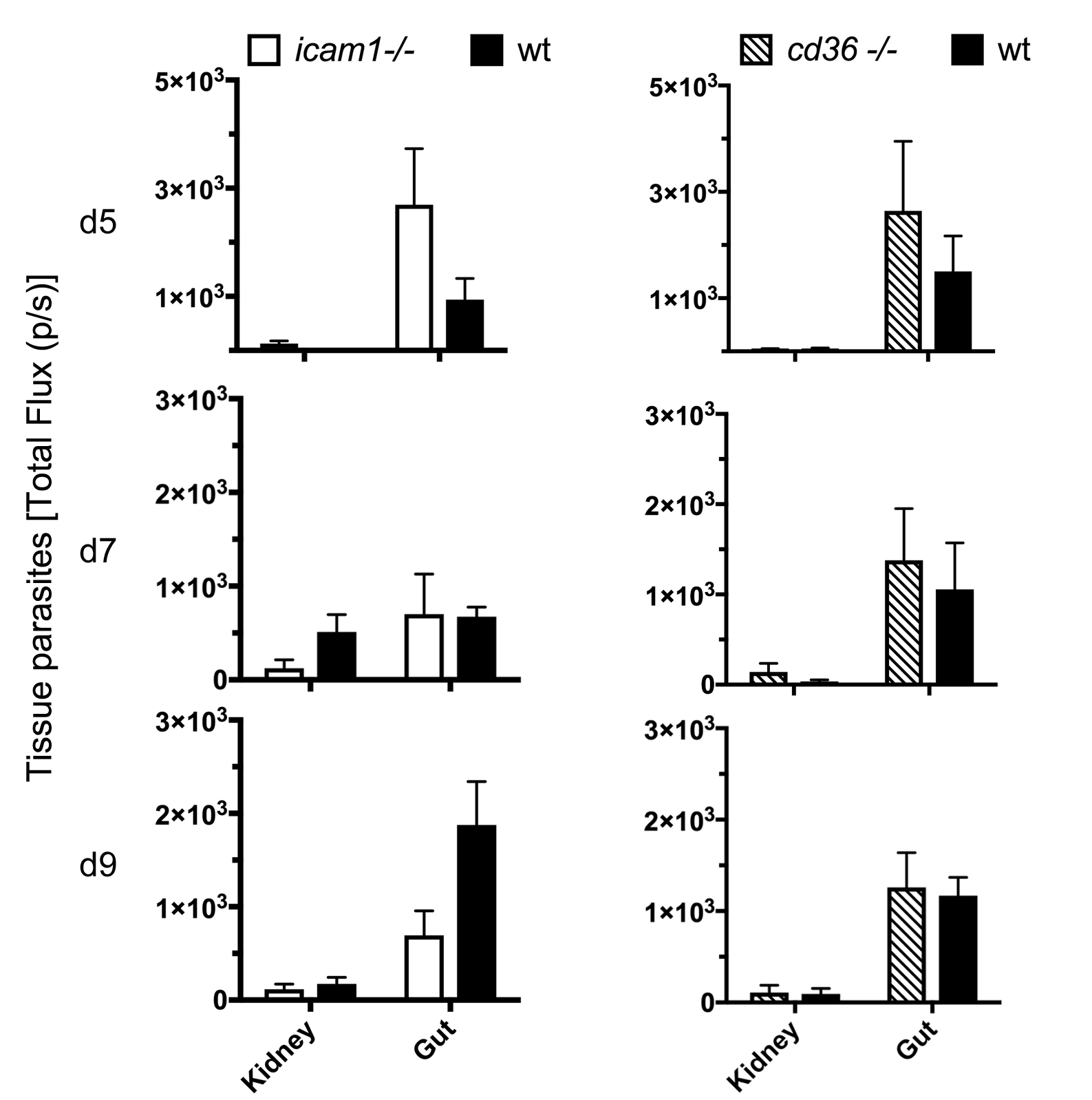

Supplement: Supplementary file 2 — Additional file 2. PCR primers for verification of integration. [file 12936_2017_1834_MOESM2_ESM.tif]

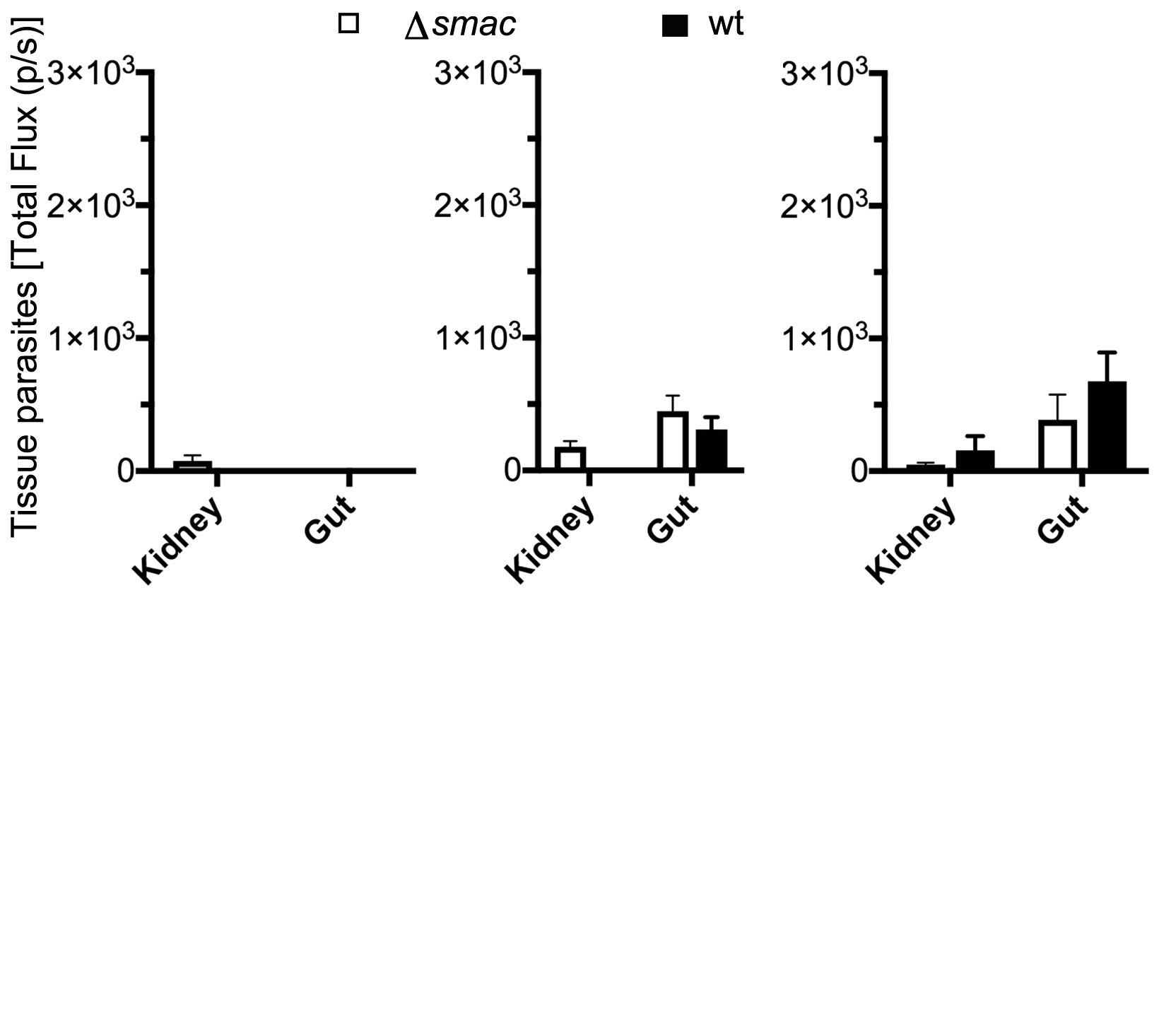

Supplement: Supplementary file 3 — Additional file 3. Similar levels of tissue parasites were seen for kidney or gut in infections of icam1−/−, cd36−/−, and their respective controls, at days 5, 7 and 9 post-infection. [file 12936_2017_1834_MOESM3_ESM.tif]
