# Supplementary material for: ICAM-1 is a key receptor mediating cytoadherence and pathology in the Plasmodium chabaudi malaria model
Source: Malar J. 2017 May 3;16:185. doi: 10.1186/s12936-017-1834-8 (PMC5415785; doi:10.1186/s12936-017-1834-8)
Supplement: Supplementary file 4 — Additional file 4. Similar levels of Δsmac and wild-type parasites accumulated in kidney and gut at days 4, 7 and 9 post-infection (n = 6). [file 12936_2017_1834_MOESM4_ESM.docx]

**PCR verification of Integration and disruption of the SMAC locus**

Amplification conditions were as follows: 94C, 5min; (95C 30s, 54C 30s, 72C 1:40) x30; 72C 7 min

| SMAC |  |  |
| --- | --- | --- |
| P1 | CGCATTATATGAGTTCATTTTAC | Internal |
| P2 | GTTATTATTTTTTATGGTGAGC | PCHAS_01_v3 52416-52437 |
| P3 | CGACATAACAAAATTGGAAAGG | PCHAS_01_v3 54645-54624 |

Integration of the plasmid was verified with primer set P1/P2 and the loss of the wild type locus shown using primer set P2/P3. Lanes 1-4 contain samples from parasites transfected with Δ *smac* (1), Δ *smacEFluc* (2), wild-type parasites (3-4), water control (Additional file 1b).
